# Supplementary figures and images for: An adapted MS2-MCP system to visualize endogenous cytoplasmic mRNA with live imaging in Caenorhabditis elegans
Source: PLoS Biol. 2024 Mar 1;22(3):e3002526. doi: 10.1371/journal.pbio.3002526 (PMC10936773; doi:10.1371/journal.pbio.3002526)

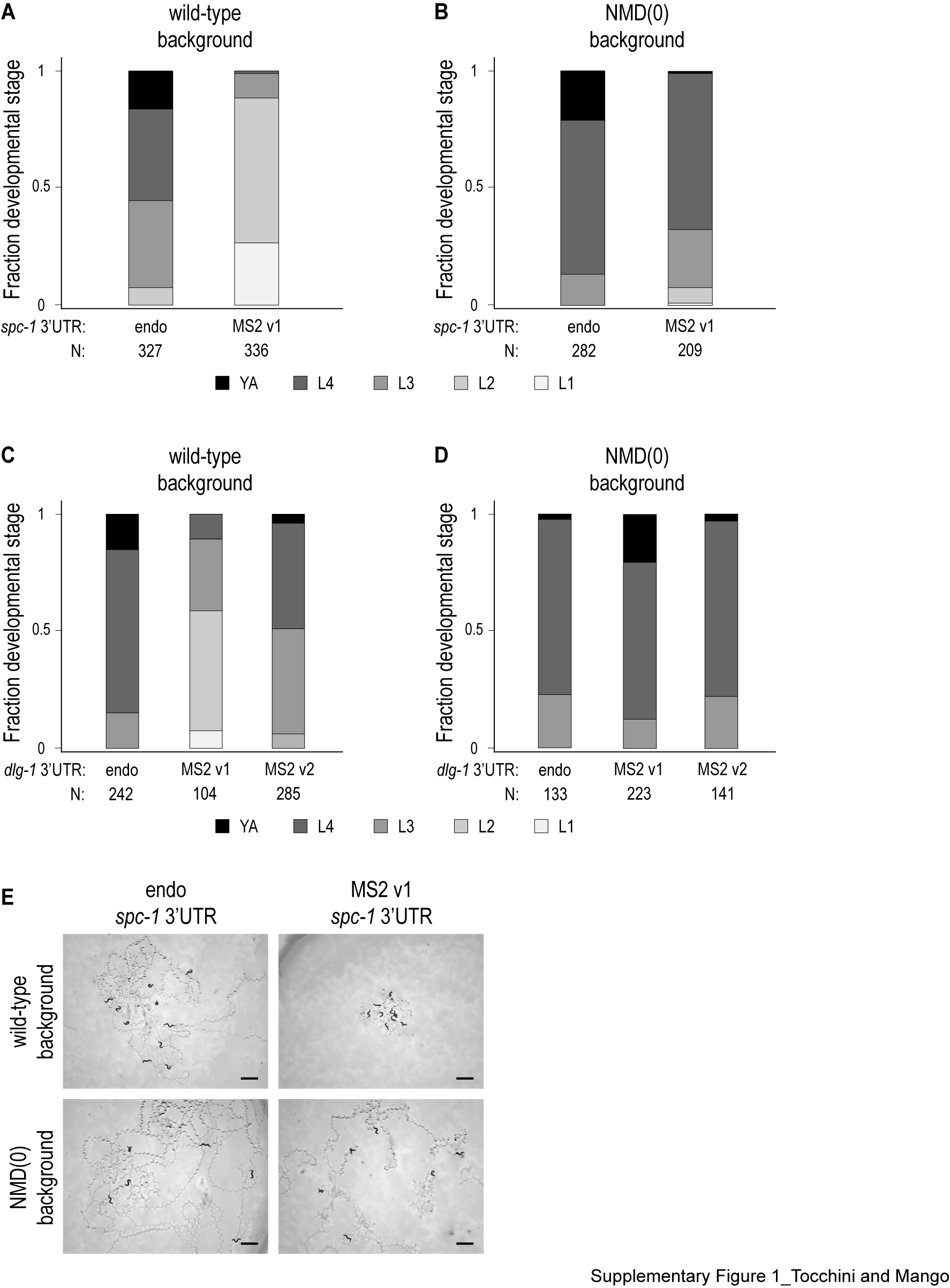

Supplement: S1 Fig — (A-D) Bar plot: in shades of grey, percentages of the different developmental stages for strains containing endogenous (endo), MS2 v1, or MS2 v2 3′ UTR for spc-1 (A, B) or dlg-1 (C, D) in a wild-type (A, C) or NMD(0) background (B, D). N values are provided. Raw data provided in S4 Table. (E) Live images of animals at the L4 stage possessing endogenous (endo, left panels) or MS2 v1 (right panels) in a wild-type (upper panels) or NMD(0) (lower panels) background. Scale bar: 1 mm. (TIF) [file pbio.3002526.s005.tif]

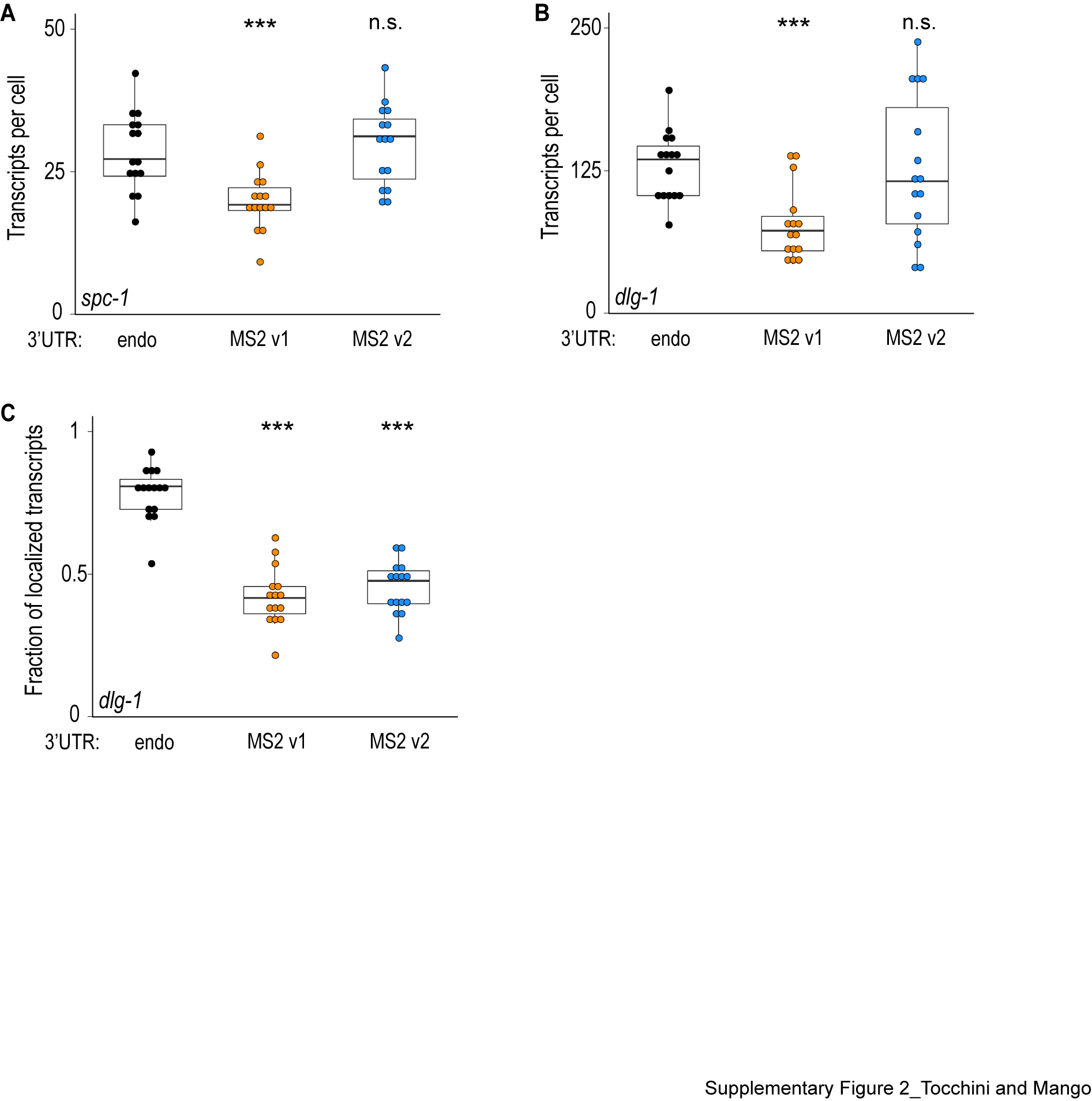

Supplement: S2 Fig — (A, B) Dot plot with box plot: Each dot represents the sum of transcripts per cell derived from the 5 most posterior seam cells of 3 comma (A) or bean (B) stage embryos with endogenous (black), MS2 v1 (orange), or MS2 v2 (blue) 3′ UTR for spc-1 (A) or dlg-1 (B) strains. Raw data provided in S4 Table. (C) Dot plot with box plot: Each dot represents the percentage of transcripts localized at the proximity of the junction [22] for endogenous (black), MS2 v1 (orange), or MS2 v2 (blue) 3′ UTR of dlg-1. The quantitation derives from the same embryos as in (B). For all these quantitation, the nuclear smFISH signal has been removed from the analysis [22] to focus on mature transcripts. Significance of statistical analyses (t test, 2 tails): n.s. > 0.05; *** < 0.001. Raw data provided in S4 Table. (TIF) [file pbio.3002526.s006.tif]

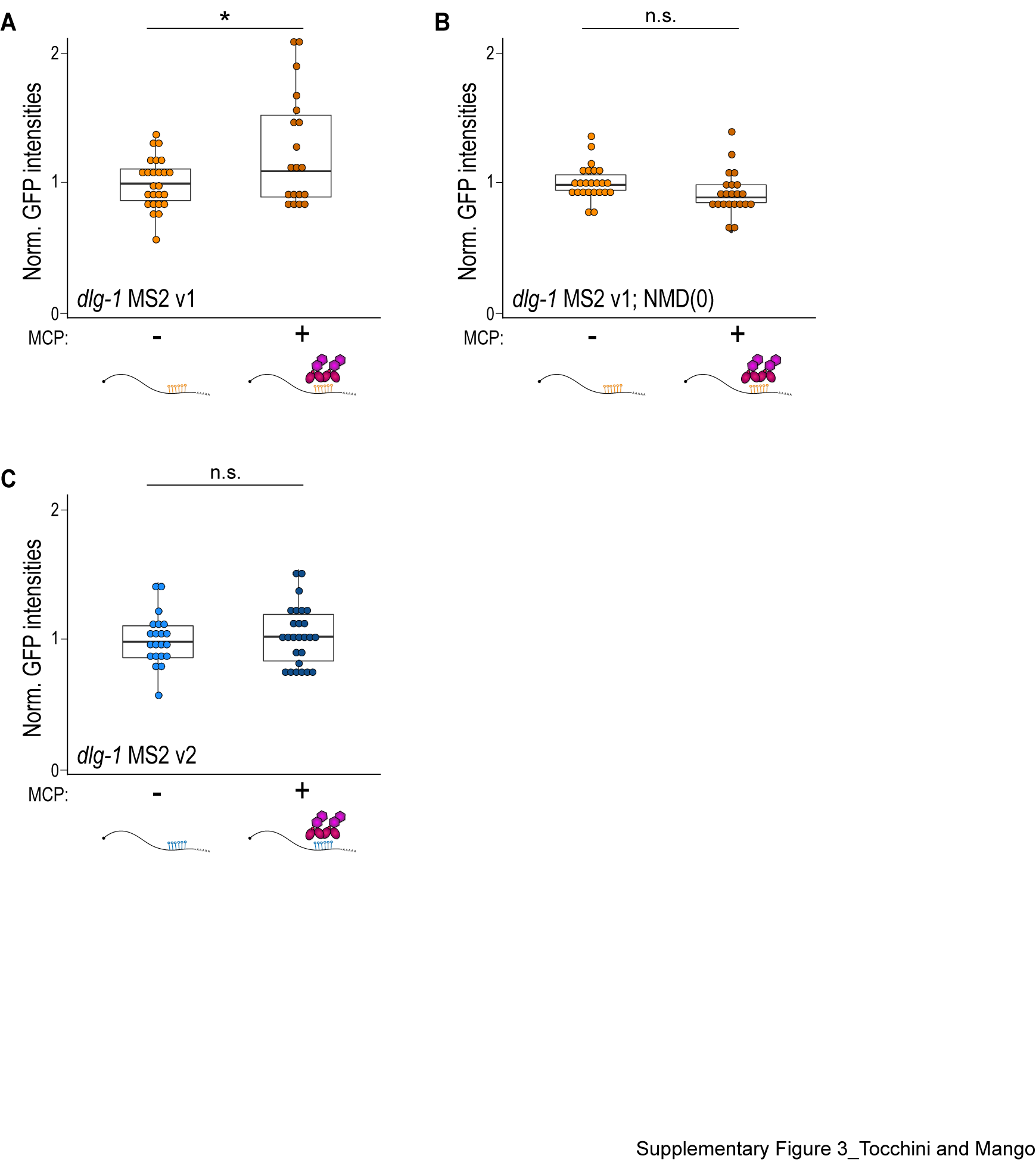

Supplement: S3 Fig — (A-C) Dot plot with box plot: Each dot represents the normalized GFP intensity of pharynges (dlg-1-tagged strain) of young adult animals (24 hours after the L4 stage) with MS2 v1 (orange) or v2 (blue) 3′ UTRs in wild-type (A, C) or NMD-deficient (B) background in the absence (“−”) or presence (“+”) of MCP. Raw data from the minus are the same as in Fig 1F (panels (A) and (C)) and Fig 2F (panel (B)) as derived from the same experiments. Significance of statistical analyses (t test, 2 tails): n.s. > 0.05; * < 0.01. Raw data provided in S4 Table. (TIF) [file pbio.3002526.s007.tif]

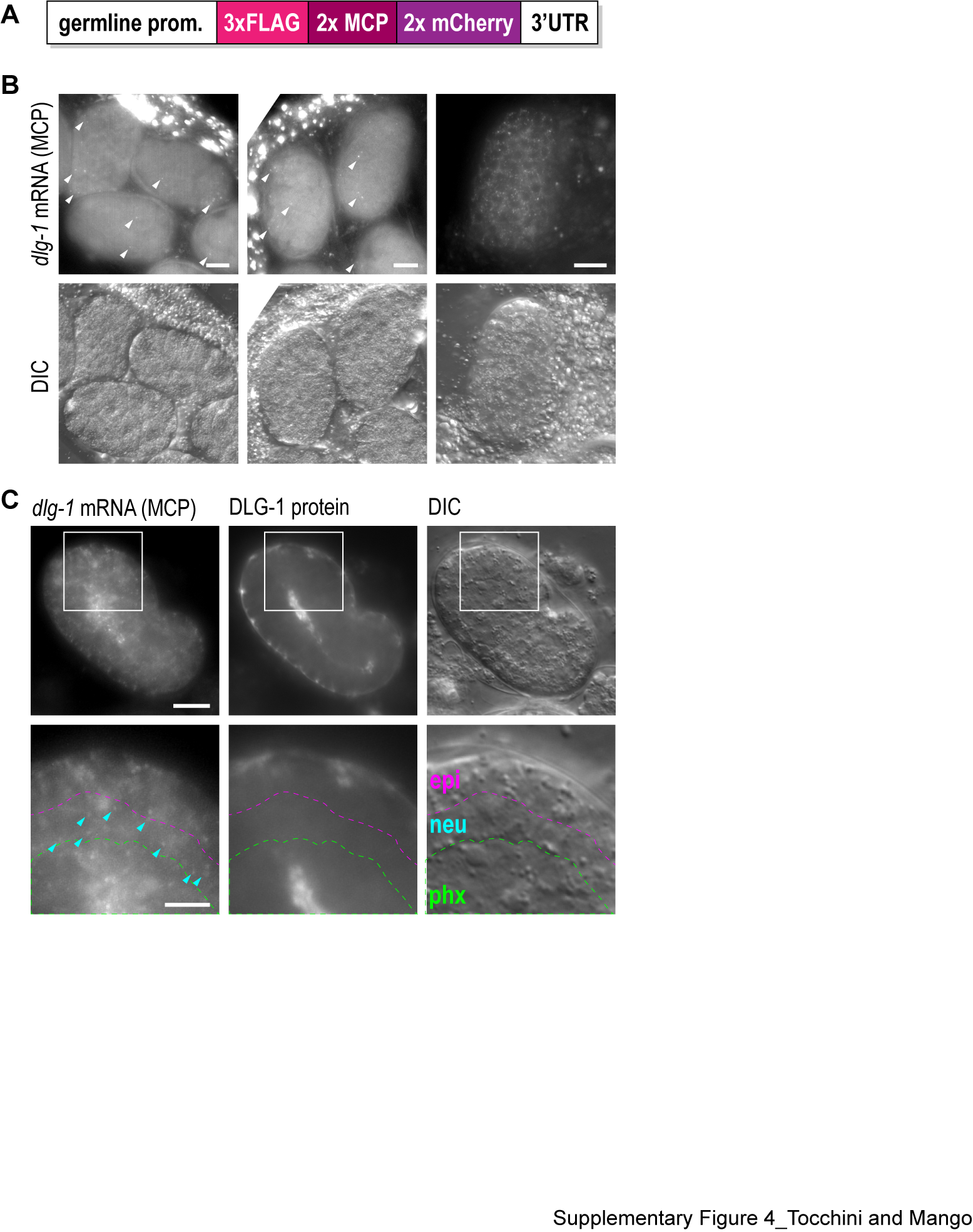

Supplement: S4 Fig — (A) Schematic representation of the MCP transgene. Two copies of MCP sequences (2x MCP) are fused to a 3xFLAG-tag sequence (3xFLAG) and 2 copies of mCherry sequences (2x mCherry). The expression of the transgene is under the control of a weak germline promoter (mesp-1p), and the 3′ UTR derives from the tbb-2 gene (3′ UTR). (B) Live fluorescent (upper panels) and DIC (lower panels) images of very early (4–26 cell-stages), early (50–100 cell-stages), mid-stage (4E) C. elegans embryos. The fluorescent images show live signal of dlg-1 MS2 v2 mRNAs visualized through the fluorescently labeled MCP. Arrowheads: examples dlg-1 mRNAs. Scale bars: 10 μm. (C) Live fluorescent (left and middle panels) and DIC (right panels) images of a comma stage C. elegans embryo (upper panels) and zoom-ins (lower panels) from the portion of the embryo highlighted in the upper panels with a white square. The fluorescent images show live signal of dlg-1 MS2 v2 mRNAs visualized through the fluorescently labeled MCP (left panels), and fluorescent signal of GFP-tagged DLG-1 protein (middle panels). Highlighted in dashed green line: developing pharynx (phx). Outside the magenta dashed line: developing epidermis (epi). In between the 2 colored dashed lines: developing neurons (neu). Cyan arrowheads: examples dlg-1 mRNAs belonging to the neuronal territory. Scale bars: 10 μm (upper panels) and 5 μm (lower panels). (TIF) [file pbio.3002526.s008.tif]
