# Supplementary material for: An adapted MS2-MCP system to visualize endogenous cytoplasmic mRNA with live imaging in Caenorhabditis elegans
Source: PLoS Biol. 2024 Mar 1;22(3):e3002526. doi: 10.1371/journal.pbio.3002526 (PMC10936773; doi:10.1371/journal.pbio.3002526)
Supplement: Attachment — Submitted filename: 137457_1_rebuttal_2755527_ryrt3v.pdf [file pbio.3002526.s010.pdf]

Revision Plan

Manuscript number: RC-2023-02047

Corresponding author(s): Cristina, Tocchini and Susan, Mango

1. General Statements [optional]

We thank the reviewers for their thoughtful comments. We were delighted that the reviewers found our manuscript and results "*solid*", "*important*", "*well-written*", "*thoughtful*", "*critical addition to the literature*", that the "*design of (these) experiments is high in quality*" and "*conclusions are convincing and the experiments are well executed*".

We were thrilled the reviewers appreciated that "*this manuscript provides solutions to technical limitations to observe mRNA* in vivo" by approaching such limitations "*in a thoughtful study where many of the salient features of MS2 epitope tagging are systematically measured*" and "*it provides a greatly improved tool to track mRNA by live imaging*" that "*also alerts of experimental noise that can be found and can be specific for each gene/transcript*"

2. Description of the planned revisions

We will address all the concerns raised by the reviewers. Most of the comments concern text edits. In addition, we will add the following to the Results section:

1. Quantitation of observed phenotypes in Figures 1C-D and 2C-D;
2. Quantitation of cytoplasmic transcripts in Figure 1G-L.

Our specific responses are shown below, in blue.

Reviewer #1

****Major comments****

The authors show that the insertion of 24xMS2 in two endogenous genes, *spc-1* and *dlg-1*, causes some phenotypes such as slow growth, lack of coordination (Unc), small body size (Sma), and reduced brood sizes. However, only an image example is provided in Fig. 1 C, D, and quantifying all these phenotypes would be nice. Same in Fig. 2C, D.

We will add the quantitation of the observed phenotypes as requested.

Similarly, the reduction in mRNA spots from smFISH in Fig. 1 G-L is difficult to visualize by eyes, and proper smFISH quantification will help interpret the results.

We will add the quantitation of the smFISH experiments as requested (as previously done in Tocchini et al., 2021).

The authors also claim a reduction in cytoplasmic RNAs and increased signal in nuclear RNAs in Fig. 1J, L. A proper quantification of nuclear and cytoplasmic smFISH will help interpret the results.

We will add the quantitation of the smFISH experiments as requested (as previously done in Tocchini et al., 2021). Regarding the quantitation of the change in nuclear signal, this is shown in Figure 3.

In Fig. 3D-F, the authors quantified the signal of nuclear smFISH. However, it is unclear to me in what samples or conditions the statistical test is performed. For example, do the three stars in Fig. 3D refer to the significant decrease or increase of NMD strain compared to the WT? What about the stars in Fig. 3E? The authors should indicate what samples they compare in the statistical test.

All the conditions are compared to the strain possessing endogenous 3'UTR in the wild-type background (Fig. 3D, left (black)). In all the instances, when a 3'UTR is in the NMD(0) background, transcription is reduced compared to when in the wild-type. We will make sure these details are clear in the text and in the figure legend.

****Minor comments****

On line 195, the authors reference Fig. 3A. However, it should be Fig. 4A.

Corrected.

In Fig. 4B, C the authors can add close to the image of the embryos the developmental stages. This will help the reader identify the embryo's developmental stage in the figure's upper and lower parts.

Added.

The authors can expand a bit the discussion on how their method differs (advantages and disadvantages) from the MASS system by Hu et al., 2023.

We will expand this section.

Reviewer #2

Major comments

They present solutions for live imaging endogenous mRNA that would be useful for colleagues interested in this technique but also show experimental noises that would be specific to each gene/transcript/UTR. In the end, the best value of this technique is to observe "real" or physiological levels but to reach this point they need to use a mutant background (NMD mutants), which may alter the "real" scenario. They found a smart way to title the article using "An adapted..." but it would be more realistic/honest to mention in the title that this is happening only in NMD mutant backgrounds. I also have doubt ion the use of the acronym MS2-MCP in the title. What about something like "Visualization of endogenous cytoplasmic mRNA with live imaging in C. elegans embryos requires an inactive Non-mediated decay"?

We used the wording “adapted” in the title to highlight the new features we enrolled in our MS2-MCP system (*i.e.*, shorter inserted MS2 sequence, lowly expressed MCP, lack on NLS in MCP). The use of an NMD-deficient background is a consequent observation that allows the constructs to be functional (unlike prior MS2-MCP configurations).

In any case, the conclusions are convincing and the experiments are well executed. I do not find the need for any essential experiments if they are clearer through the manuscript (from title and abstract to discussion) that this technique may need to be optimized and (and maybe validated with FISH) for each specific transcript, and developmental stage cell type where NMD and polyadenylation may work differently.

We added a note in the discussion.

Another source of experimental noise may come from the use of mCherry, which is known for forming aggregates in some cells/stages. (would this artefactual aggregation occur in figure 1L?).

In Figure 1, the mCherry-tagged MCP is not present in the background. In Figure 1, only the MS2 insertions have been analyzed.

The only experiment that I missed, not essential but easy to perform, is a better description of the slight developmental delay of dlg-1 MS2 v2 animals. Size measures? Time until they lay embryos?

We will add the quantitation of the observed phenotypes as requested.

In this sense, although is not the main purpose of the article, they could highlight the fact that this is an additional option to produce hypomorphic alleles of essential genes.

Added.

Lines 236-237: "*Upon MS2 insertion loss-of-function phenotypes appear, and such a notion can be potentially applied to generate hypomorphic alleles*."

Regarding methods, I miss information about the CRISPR-Cas efficiency of inserting the MS2 sequences at the UTRs. Sizes are "small" and can facilitate the insertion of dsDNA repair template, but it would be useful to know what efficiency would be expected.

Added.

Lines 320-323: "*Specifically, 20 young adult animals were injected. From these, around 20 F1s with a Rol phenotype were singled. The following number of positive insertions were obtained for spc-1 and dlg-1: 2/22 for spc-1 MS2 v1; 5/24 for spc-1 MS2 v2; 1/24 for dlg-1 MS2 v1; 2/18 for dlg-1 MS2 v2*."

It would be good to mention somewhere how frequent are GG PAM sequences at UTRs sequences (probably less common than in other regions). In this sense, the use of minimal PAM Cas9 variants (Vicencio et al, Nat Comm 2022) would be necessary.

Indeed, genomic 3'UTR sequences are enriched in A-T base pairs, whereas coding regions may possess more C-G base pairs. This difference implies a higher chance of finding a PAM site in an open reading frame (ORF). Nevertheless, inserting a sequence in an ORF frequently represents a challenge, not only because the frame of translation needs to be maintained, but also because of the restriction given by the location where the sequence needs to be inserted (*e.g*., amino- or carboxy-terminus). All in all, inserting a non-coding sequence (MS2 hairpins) in a UTR is much simpler than inserting a coding sequence in an ORF: the putative lower abundance of PAM sites in a 3'UTR is compensated by the lack of need to maintain the translation frame and the absence of restriction for a precise position.

We will discuss this issue in the Discussion and add the suggested reference.

Minor comments

In the abstract, line 33, remove epithelial? I do not think this is relevant in this sentence.

We prefer to keep it as we specifically looked at epithelial cells.

In figure 4, panels B and C, add the two different embryonic stages on the left side. Then, it wouldn't be necessary to read the legend to understand the figure.

Added.

Revision Plan

Reviewer #3

Major comments

-

Minor comments

The authors should provide more details in the experimental description of the MS2-tagged alleles (or in the figure images). It needs to be clarified in the main text how many MS2 hairpins there are, though this can be found in the materials and methods. In addition, it would be nice to know if these were any of the variations of MS2 hairpins that have already been optimized in some other way to increase or decrease structure or RNA metabolism defects in other systems. Specifically, are these hairpins the newest versions, V6 or V7, described in manuscripts from the Singer laboratory (e.g., (Tutucci et al., 2018))? For aficionados of this imaging system, it would be important to qualify each of the potential new features that make the results in this manuscript so clear and important.

Added.

Lines 315-318: "*To reduce the overall size of the 24xMS2 hairpin sequence the number of linker nucleotides between the hairpins was reduced from 30 (Wu et al., 2015) to 3 nucleotides, resulting in the overall length of the MS2 insert from 1,275 to 634 nucleotides (Jeffrey Chao, personal communication)*."

For people that are colorblind (or have reduced ability to distinguish some colors from others (like me, a reviewer)), it would be nice to have the MS2 illustrations in Figures 1A and B not have that color within the black, normal UTR. It's picky, but I had to ask someone what color that was.

Corrected. We apologize for the mistake and thanks for pointing it out.

3. Description of the revisions that have already been incorporated in the transferred manuscript

Some of the textual changes have already been made, please see points above.

4. Description of analyses that authors prefer not to carry out

We will not undertake an analysis of PAM frequency in 3'UTRs, but instead comment regarding how to work around PAM requirements in the text and Discussion.
