# Supplementary material for: An adapted MS2-MCP system to visualize endogenous cytoplasmic mRNA with live imaging in Caenorhabditis elegans
Source: PLoS Biol. 2024 Mar 1;22(3):e3002526. doi: 10.1371/journal.pbio.3002526 (PMC10936773; doi:10.1371/journal.pbio.3002526)
Supplement: Attachment — Submitted filename: Response to Reviewers.pdf [file pbio.3002526.s012.pdf]

To the Editors at PLOS Biology,

We thank the reviewers for their thoughtful comments. We were delighted that the reviewers found our manuscript and results "*important*", "*well-written*", "*thoughtful*", a "*critical addition to the literature*", and that they stated that the "*design of (these) experiments is high in quality*," the "*conclusions are convincing and the experiments are well executed*". We note that the reviewers also remarked that this manuscript *"provides a greatly improved tool to track mRNA by live imaging."*

We have addressed all of the concerns raised by the Reviewers and Editor, including text edits, and the addition of three experiments:

1. Quantitation of observed phenotypes in Figures 1C-D and 2C-D (Figure S1);
2. Quantitation of cytoplasmic transcripts in Figure 1G-L (Figure S2);
3. Visualization of transcripts with our MS2-MCP system in the early embryo.

We have revised our writing throughout to clarify the text, in response to queries from the reviewers particularly R2. Our specific responses are shown below, in blue.

Reviewer #1

Major comments

The authors show that the insertion of 24xMS2 in two endogenous genes, *spc-1* and *dlg-1*, causes some phenotypes such as slow growth, lack of coordination (Unc), small body size (Sma), and reduced brood sizes. However, only an image example is provided in Fig. 1 C, D, and quantifying all these phenotypes would be nice. Same in Fig. 2C, D.

We have added the quantitation of the observed phenotypes in Figure S1.

Similarly, the reduction in mRNA spots from smFISH in Fig. 1 G-L is difficult to visualize by eyes, and proper smFISH quantification will help interpret the results.

We have added the quantitation of the smFISH experiments in Figure S2.

The authors also claim a reduction in cytoplasmic RNAs and increased signal in nuclear RNAs in Fig. 1J, L. A proper quantification of nuclear and cytoplasmic smFISH will help interpret the results.

We have added the quantitation of the smFISH experiments in Figure S2. Regarding the quantitation of the change in nuclear signal, this is shown in Figure 3.

In Fig. 3D-F, the authors quantified the signal of nuclear smFISH. However, it is unclear to me in what samples or conditions the statistical test is performed. For example, do the three stars in Fig. 3D refer to the significant decrease or increase of NMD strain compared to the WT?

What about the stars in Fig. 3E? The authors should indicate what samples they compare in the statistical test.

The original statistics referred to the strain possessing the endogenous 3'UTR in the wild-type background (Fig. 3D, left (black)), and we have colored them now in magenta. We have also added, in black, the statistics comparing the different 3'UTR strains between wild-type and NMD(0) backgrounds. In the corresponding figure legend, we clarified:

Statistics in black: comparison of each 3'UTR (endo, MS2 v1, and MS2 v2) in wild-type versus NMD(0) background. Statistics in magenta: comparison of each strain to the reference one carrying and endogenous 3'UTR in the wild-type background.

Minor comments

On line 195, the authors reference Fig. 3A. However, it should be Fig. 4A.

Corrected.

In Fig. 4B, C the authors can add close to the image of the embryos the developmental stages. This will help the reader identify the embryo's developmental stage in the figure's upper and lower parts.

Added.

The authors can expand a bit the discussion on how their method differs (advantages and disadvantages) from the MASS system by Hu et al., 2023.

We have expanded this section in a separate paragraph of the discussion with the title *"Previous methods to visualize RNA live in C. elegans".*

Reviewer #2

Major comments

They present solutions for live imaging endogenous mRNA that would be useful for colleagues interested in this technique but also show experimental noises that would be specific to each gene/transcript/UTR. In the end, the best value of this technique is to observe "real" or physiological levels but to reach this point they need to use a mutant background (NMD mutants), which may alter the "real" scenario. They found a smart way to title the article using "An adapted..." but it would be more realistic/honest to mention in the title that this is happening only in NMD mutant backgrounds. I also have doubt ion the use of the acronym MS2-MCP in the title. What about something like "Visualization of endogenous cytoplasmic mRNA with live imaging in C. elegans embryos requires an inactive Non-mediated decay"?

We used the wording "adapted" in the title to highlight the new features we designed (*i.e.*, shorter inserted MS2 sequence, lowly expressed MCP, lack of an NLS in MCP, incorporation

of NMD). The use of an NMD-deficient background is a consequent observation that allows the constructs to be functional (unlike prior MS2-MCP configurations).

In the abstract, we state that the inactivation of the nonsense-mediated decay pathway is key to observing RNAs with MS2 hairpins inserted in their 3'UTRs. We suspect readers might like to know the paper concerns MS2-MCP, but we will leave it up to the editors to decide which title they prefer.

In any case, the conclusions are convincing and the experiments are well executed. I do not find the need for any essential experiments if they are clearer through the manuscript (from title and abstract to discussion) that this technique may need to be optimized and (and maybe validated with FISH) for each specific transcript, and developmental stage cell type where NMD and polyadenylation may work differently.

We have added in the Discussion the following:

When tagging a new mRNA with MS2 hairpins, we therefore recommend considering the site of insertion, validation with smFISH, possible differences in poly(A) sites usage, and differences in the NMD(0) background in the studied cell type(s).

Another source of experimental noise may come from the use of mCherry, which is known for forming aggregates in some cells/stages. (would this artefactual aggregation occur in figure 1L?).

No. In Figure 1, the mCherry-tagged MCP is not present. In Figure 1, only the MS2 tagged RNAs are present in the analyzed strains.

The only experiment that I missed, not essential but easy to perform, is a better description of the slight developmental delay of dlg-1 MS2 v2 animals. Size measures? Time until they lay embryos?

We have added the quantitation of the observed phenotypes in Figure S1.

In this sense, although is not the main purpose of the article, they could highlight the fact that this is an additional option to produce hypomorphic alleles of essential genes.

We have added a paragraph in the Discussion which also includes this point. Specifically, we added:

Likewise, MS2 insertions produce loss-of-function phenotypes and could be used as a strategy to generate hypomorphic alleles.

Regarding methods, I miss information about the CRISPR-Cas efficiency of inserting the MS2 sequences at the UTRs. Sizes are "small" and can facilitate the insertion of dsDNA repair template, but it would be useful to know what efficiency would be expected.

Added:

Specifically, 20 young adult animals were injected. From these, around 20 F1s with a Rol phenotype were singled. The following number of positive insertions were obtained for spc-1 and dlg-1: 2/22 for spc-1 MS2 v1; 5/24 for spc-1 MS2 v2; 1/24 for dlg-1 MS2 v1; 2/18 for dlg-1 MS2 v2.

It would be good to mention somewhere how frequent are GG PAM sequences at UTRs sequences (probably less common than in other regions). In this sense, the use of minimal PAM Cas9 variants (Vicencio et al, Nat Comm 2022) would be necessary.

Indeed, genomic 3'UTR sequences are enriched in A-T base pairs, whereas coding regions possess more C-G base pairs. This difference implies a higher chance of finding a PAM site in an open reading frame (ORF). Nevertheless, inserting a sequence in an ORF frequently represents a challenge, not only because the frame of translation needs to be maintained, but also because of the restriction given by the location where the sequence needs to be inserted (*e.g.*, amino- or carboxy-terminus). All in all, inserting a non-coding sequence (MS2 hairpins) in a UTR is much simpler than inserting a coding sequence in an ORF: the putative lower abundance of PAM sites in a 3'UTR is compensated by the lack of need to maintain the translation frame and the absence of restriction for a precise position.

We added the following sentence when describing how to insert the MS2 sequence in Materials and Method:

Given the low percentage of GCs in 3'UTR, users may want to consider Cas9 variants with minimal PAM sequences as an alternative method to insert the MS2 sequence (Vicencio et al., 2022).

Minor comments

In the abstract, line 33, remove epithelial? I do not think this is relevant in this sentence.

We removed it, given that we have now looked at other cell types too.

In figure 4, panels B and C, add the two different embryonic stages on the left side. Then, it wouldn't be necessary to read the legend to understand the figure.

Added.

Reviewer #3

Major comments

-

Minor comments

The authors should provide more details in the experimental description of the MS2-tagged alleles (or in the figure images). It needs to be clarified in the main text how many MS2 hairpins

there are, though this can be found in the materials and methods. In addition, it would be nice to know if these were any of the variations of MS2 hairpins that have already been optimized in some other way to increase or decrease structure or RNA metabolism defects in other systems. Specifically, are these hairpins the newest versions, V6 or V7, described in manuscripts from the Singer laboratory (e.g., (Tutucci et al., 2018))? For aficionados of this imaging system, it would be important to qualify each of the potential new features that make the results in this manuscript so clear and important.

Added:

To reduce the overall size of the 24xMS2 hairpin sequence the number of linker nucleotides between the hairpins was reduced from 30 (Wu et al., 2015) to 3 nucleotides, resulting in the overall length of the MS2 insert from 1,275 to 634 nucleotides (Jeffrey Chao, personal communication).

For people that are colorblind (or have reduced ability to distinguish some colors from others (like me, a reviewer)), it would be nice to have the MS2 illustrations in Figures 1A and B not have that color within the black, normal UTR. It's picky, but I had to ask someone what color that was.

Corrected. We apologize for the mistake and thanks for pointing it out.

Academic editor

Personally, I do not consider the NMD mutant background a major problem. By itself, it is an interesting finding that apparently the NMD system can identify such features. Also in other systems, effects of the NMD system may be present, but they may have gone undetected (even if the method as such seems to work in a wild-type NMD background, NMD may affect the precise outcome...I guess this is not known-can be addressed in discussion perhaps?).

We have expanded this topic in the first paragraph of the Discussion.

What I consider a bigger issue, not addressed by the reviewers it seems, is that the system is only tested in epithelial cells. For a method paper I would expect to see it also probed in another tissue. Germline would be a good choice, or early embryo.

The *hsp-16.48* promoter we used to drive low expression of MCP is not expressed in the germ line or early embryo. To address the Editor’s concern, we re-engineered the MCP transgene and placed it under the control of a low expressed germline promoter, *mesp-1p* (Aljohani et al., 2023). In this way, MCP is now present in germ cells and all embryonic stages. We show that MCP detects RNAs in early embryos, in the gut, and in neurons, greatly expanding the list of tissues (Figure S4).

To note: the computer processing that we normally use on smFISH data is down. Rather than hold up the paper any longer, we have decided to submit the figures as is. If the reviewers or editors want a cleaned up version, we are happy to try to do this once OMERO is working again.

One phrase in the discussion confused me; the authors write:

“We speculate that binding of MCP to the MS2-tagged mRNA might protect the mRNA from NMD-mediated degradation in a wild-type background. On the other hand, when the NMD pathway is inhibited, MCP might have a marginal role in posttranscriptional gene regulation via stabilizing the mRNA resulting in a higher protein output per transcript and overall.”

In both wildtype and mutant NMD conditions this explanation seems to go towards a stimulatory role for MCP. However, the authors write in the sentence before this phrase:'...in an NMD(0) background MCP led to a decrease.' This is inconsistent. I guess this is a textual error, but has to be corrected, or clarified.

Indeed, it was a textual error, and we have corrected the last sentence as follows:

On the other hand, when the NMD pathway is inhibited, MCP might have a marginal role in post-transcriptional gene regulation possibly via translational repression which results in a lower protein output per transcript and overall.
